# Emergence of *bla*_NDM-5_-Carrying Klebsiella aerogenes in Japan

**DOI:** 10.1128/spectrum.02222-21

**Published:** 2022-06-06

**Authors:** Shizuo Kayama, Liansheng Yu, Sayoko Kawakami, Koji Yahara, Junzo Hisatsune, Masaru Yamamoto, Keiko Yamamoto, Nobuyuki Shimono, Yasushi Kibe, Makiko Kiyosuke, Motoyuki Sugai

**Affiliations:** a Antimicrobial Resistance Research Centre, National Institute of Infectious Diseases, Higashimurayama City, Tokyo, Japan; b Project Research Center for Nosocomial Infectious Diseases, Hiroshima University, Hiroshima City, Hiroshima, Japan; c Department of Antimicrobial Resistance, Hiroshima University Graduate School of Biomedical & Health Sciences, Hiroshima City, Hiroshima, Japan; d Central Clinical Laboratory, Toyohashi Municipal Hospital, Toyohashi, Aichi, Japan; e Center for the Study of Global Infection, Kyushu University Hospital, Fukuoka, Japan; f Department of Clinical Chemistry and Laboratory Medicine, Kyushu University Hospital, Fukuoka, Japan; Public Health Ontario

**Keywords:** *bla*_NDM-5_, *Klebsiella aerogenes*, carbapenems

## LETTER

An increase in carbapenemase-producing *Enterobacterales* is the most serious risk to public health among drug-resistant bacteria. Since 2018, Klebsiella aerogenes has been ranked among the top 35 to 40% of carbapenem-resistant *Enterobacteriaceae* (CRE) isolated in Japan (1, 2), but few carbapenemase-positive isolates have been reported (3, 4). In the Japan Antimicrobial Resistant Bacterial Surveillance conducted in 2019 to 2020, 1,725 *Enterobacterales* isolates showing meropenem (MEM) MICs of ≥0.25 μg/mL were collected from 175 medical institutions throughout Japan. In this surveillance, there were 142 K. aerogenes isolates collected with low susceptibility to carbapenem, which was similar to the number of Escherichia coli isolates collected (146 isolates). Among the K. aerogenes isolates, 97.2% of them did not have carbapenemases. Two *bla*_NDM-5_-carrying K. aerogenes strains were isolated in 2 regions that are 800 km apart (Fukuoka and Aichi Prefectures). The Aichi and Fukuoka isolates were isolated from vaginal discharge and spontaneous urination, respectively. Both isolates were resistant to imipenem (IPM)/MEM (MIC of >8 μg/mL).

**FIG 1:**
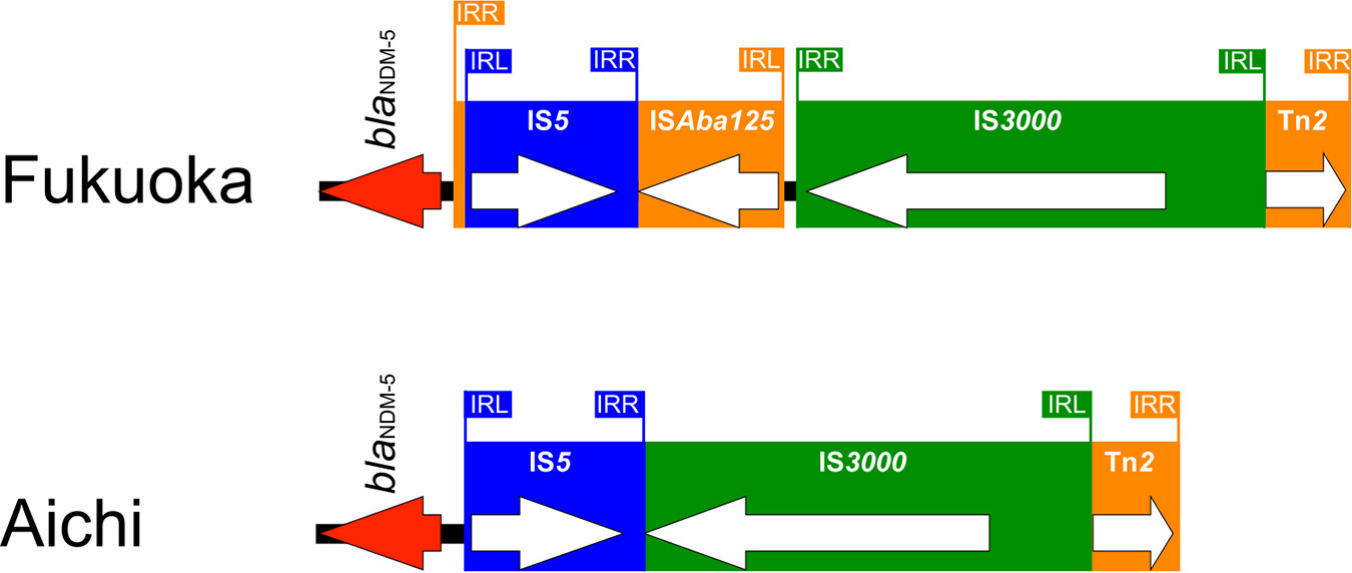
Detailed comparison of the 5′ ends of *bla*_NDM-5_ sequences of the K. aerogenes NDM-5 plasmid of the Fukuoka isolate (accession number DRA011229) and of the Aichi isolate (accession number DRA011229) from Japan. Red arrows and white arrows in blue and green boxes indicate *bla*_NDM-5_, IS*5*, and IS*3000*, respectively.

Complete sequences of the Fukuoka and Aichi plasmids obtained by short-read sequencing with Illumina and long-read sequencing with MinION revealed that *bla*_NDM-5_ was present on the IncX3 plasmid of 46,161 bp and 44,811 bp, respectively. A linear comparison of *bla*_NDM-5_ plasmid sequences revealed that these plasmids are similar to those from China: pNDM-Z244, pP855-NDM5, and pNDM-CR33 (accession numbers MK450346, MF547508, and MK450349, respectively). No other resistance genes were detected in these plasmids using ResFinder 3.2. In both the Fukuoka and Aichi plasmids, Tn*2* inserted upstream of *bla*_NDM-5_ was inactivated by an insertion of IS*3000* (Fig. 1). In the Fukuoka plasmid, IS*Aba125* just upstream of *bla*_NDM-5_ was disrupted by insertion of IS*5*, while in the Aichi plasmid IS*5* was inserted at the 3′ portion of IS*3000*. Although IS*5* is present in both plasmids in similar positions, the different insertion order appears to suggest different origins of the plasmids (Fig. 1).

Analysis by MLST-2.0 showed different chromosomal backgrounds, with ST209 in the Fukuoka isolate, and the new sequence type ST224 (nearest STs: 10, 117, and 12) in the Aichi isolate. In addition to the *bla*_NDM-5_ plasmid, the Aichi strain also carried 3 other plasmids. One of them is 116,630 bp and contained *aadA16*, *aac(3)-IId*, *aph(6)-Id*, *aph(3″)-Ib*, *aac(6′)-Ib-cr*, *mph(A)*, *sul1*, *sul2*, *qnrB52*, *qnrB2*, *qnrS1*, *bla*_TEM-1B_, *bla*_CTX-M-3_, *ARR-3*, *tet(A)*, *floR*, and *dfrA27*. The other two plasmids were small and did not carry drug resistance genes. In the Fukuoka isolate, the *bla*_NDM-5_ plasmid was the only completed one, and the sequence reads converged to 6 contigs. These nucleotide fragments together with the plasmid indicated that the Fukuoka isolate had no resistance genes other than the *bla*_NDM-5_ in the plasmid and *fosA7* in the chromosome.

A BLAST search revealed 23 previous reports of *bla*_NDM_-carrying K. aerogenes isolates around the world, with a *bla*_NDM-5_-carrying isolate reported in China in 2020 (5). Japanese cases had no history of traveling abroad, thereby suggesting that there is no epidemiological link with China.

The emergence of K. aerogenes isolates carrying the *bla*_NDM-5_ plasmid should henceforth call for more careful attention to carbapenemase-producing K. aerogenes.

### Data availability.

The nucleotide sequence of the NDM-5-carrying K. aerogenes isolate described in this study was deposited in the DDBJ Sequence Read Archive (DRA) under accession number DRA011229 (BioSample SAMD00261449 for Fukuoka isolate JARBS-GNR_440044-19-0003 and SAMD00261448 for Aichi isolate JARBS-GNR_23029-19-0094).
